# Monitoring Heavy Metal Contents with *Sphagnum Junghuhnianum* Moss Bags in Relation to Traffic Volume in Wuxi, China

**DOI:** 10.3390/ijerph15020374

**Published:** 2018-02-22

**Authors:** Rong Hu, Yun Yan, Xiaoli Zhou, Yanan Wang, Yanming Fang

**Affiliations:** Co-Innovation Center for Sustainable Forestry in Southern China, College of Biology and the Environment, Nanjing Forestry University, Nanjing 210037, China; hurong809228@163.com (R.H.); yanyun3100090@163.com (Y.Y.); zhouxiaoli0404@163.com (X.Z.); m13770762069@163.com (Y.W.)

**Keywords:** heavy metal, moss bag method, *Sphagnum junghuhnianum*

## Abstract

Despite its small size, a moss bag can reveal the different temporal and spatial deposition patterns of pollutants at a particular site; therefore, researchers can use moss bags to determine pollution sources and to put forward strategies for pollution control. Although the use of moss bags to monitor atmospheric pollution has been widely reported in Europe, there are few such empirical studies in China. Thus, in this study, bags containing the moss *Sphagnum junghuhnianum* were used to assess the concentrations of heavy metals (chromium (Cr), copper (Cu), lead (Pb), vanadium (V), and zinc (Zn)) at five sampling sites (four roads and a forest park) during the summer and winter of 2012. According to the relative accumulation factor (RAF) and contamination factor (CF) results, pollution in winter was heavier than that in summer, and Cr was found to be the most contaminating, having the highest mean CF. There was a significant positive correlation (*p* < 0.05) between traffic volume and concentration for three heavy metals (Cr, Cu, and V) in winter, whereas a significant positive correlation (*p* < 0.05) was observed between traffic volume and concentrations for four heavy metal elements (Cr, Pb, V, and Zn) in summer, indicating a close relationship between heavy metal contents and traffic volume. Although there was substantial variation in the concentrations of the five heavy metals in the moss bags, significant correlations between heavy metals suggested that the contaminants originated from a common source, namely vehicle emissions. The results demonstrated that the four roads were subject to different degrees of pollution depending on the volume of traffic using each road. Therefore, the results of this study suggest that traffic volume is a major reason for heavy metal pollution.

## 1. Introduction

Traffic pollution is one of the most important sources of environmental pollution in urban areas, especially when heavy industry is not present. Research shows that air pollution caused by traffic is becoming increasingly prominent, with soot-type pollution in some cities being replaced by pollution from motor vehicle emissions. The U.S. Environmental Protection Agency (EPA) investigated contaminants resulting from vehicle emissions, including heavy metals, such as Cr, Cu, Pb, V, and Zn [1], and found a direct link between air pollution and traffic [2,3]. In particular, heavy metal pollution results from motor vehicle exhaust emissions, secondary dust raised by vehicles, wear of tyres and other parts of vehicles, and corrosion [4,5,6,7,8]. Heavy metals are ingested via the respiratory or digestive tracts into the human body, causing serious harm to human health. Therefore, increasing research attention has been paid to heavy metal air pollution. However, existing air pollution monitoring methods are expensive and difficult to perform. As a result of its simple structure, strong adsorption capacity, and easy access compared with other biosorbents, mosses have a significantly higher concentration of surface groups that are able to bind such metals at the cell surface [9], which explains why they can be used to monitor such elements. Moss has also been used as an ideal biological indicator for atmospheric environmental monitoring. The use of moss bags to monitor environmental pollution originated in Europe, where it is now widely used, as reviewed by Onianwa [10]. The moss bag technique was originally introduced by Goodman and Roberts and later modified by Little and Martin. Goodman and Roberts [11] used *Hypnum cupressiforme* to monitor the concentration of Zn, Ni, Pb, and Cr in an industrial area in southwest Wales, whereas Cameron and Nickless [12] used the moss bag method to monitor Zn, Pb, and Cr concentrations near a lead-zinc smelter in Britain. Moss bags have also been widely used to monitor atmospheric pollutants in many countries, including Finland [13,14,15], the USA [16], Bulgaria [17], Italy [18,19], the Slovak Republic [20], Poland [21], and Serbia [22,23,24,25]. The most influential of these was a 5-year European transnational joint monitoring program involving more than 100 scientists from 28 countries [2,26,27,28]. In China, use of the moss bag method to determine pollution levels has been documented in Shanghai [29], Chongqing [30], and Guizhou [31]. Moss bags can be used to monitor not only urban air pollution, but also air around volcanoes [32], tunnels [33], airports [34], and bodies of water [35].

Wuxi city is located at E119°31′~120°36′, N31°07′~32°02′ in southern Jiangsu Province, which covers an area of approximately 4628 km^2^. It has a typical subtropical humid climate, with a mean temperature of 1.2 °C in January and 31.2 °C in July, and a mean annual precipitation of 1121 mm. Wuxi is an important industrial city located in the Yangtze River Delta. Along with rapid economic development in recent years, urban construction in Wuxi has also increased rapidly, as has motor vehicle ownership. The number of vehicles registered in the city had reached 573,000 by the end of 2011, and will continue to increase at an annual rate of 15% according to local statistics.

Therefore, the objectives of the current study were to use moss bags to: (1) evaluate the degree of pollution of heavy metals (Cr, Cu, Pb, V, and Zn) at four roads in Wuxi; (2) determine the difference in heavy metal concentrations between the winter (low temperatures and low rainfall) and the summer (high temperatures and high rainfall); (3) analyze the influence of traffic volume on the airborne concentration of heavy metals; and (4) analyze the relationships among the five heavy metal elements at each study site.

## 2. Materials and Methods

### 2.1. Moss Sampling, Bag Preparation and Exposure

*Sphagnum junghuhnianum* moss was collected from Longwangshan, Anji, Zhejiang Province. The moss is easy to identify. It is also easy to handle in the laboratory and has been used repeatedly in other biomonitoring studies with moss bags. Moss carpets were collected from the ground in open spaces, cleaned with water, and air-dried. Moss bags were prepared by weighing out 2 g of moss (air-dried weight) and packing it loosely in nylon nets of 18 × 14 cm with a mesh size of 2 mm^2^.

**Figure 1:**
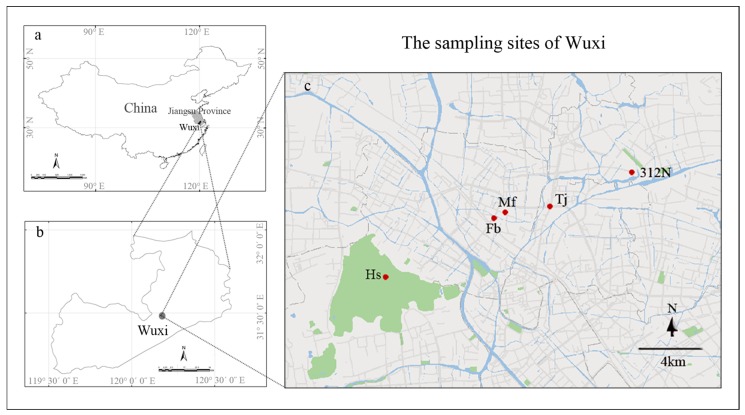
Location of Wuxi in China (**a**); location of study area in Wuxi (**b**); and a map of Wuxi showing the five monitoring sites: four roads (Mf: Minfeng Road, Fb: Fengbin Road, 312N: 312 National Road, Tj: Tongjiang Avenue) and a control site (Hs: Huishan) (**c**).

The moss bags were set up at five study sites (Figure 1): one control site at Huishan National Forest Park (Huishan), and four at different roads (Minfeng, Fengbin, 312 National Road, and Tongjiang Avenue). Minfeng connects several residential districts, while Fengbin is located at the junction of Fengxiang Road with Xicheng Road. 312 National Road passes through Wuxi, and runs for 48.43 km. Tongjiang Avenue is connected to the Shanghai-Nanjing and Xicheng Expressways, which are important roads for travel in and out of the city. There are no other sources of pollution around the four selected roads; thus, we could be certain that any heavy metal elements detected in the bags would result from traffic pollution rather than from other sources.

The moss bags were exposed to the air by hanging them approximately 3–4 m above the ground at the monitoring sites for 6 weeks [33]. Each bag was fully exposed to the air. Five bags were placed at each monitoring site (including the control site) and left in place for 6 weeks from 8 January to 29 February 2012, and from 27 May to 8 July 2012 [36]. The moss bags were then taken to the laboratory for chemical analysis.

### 2.2. Traffic Census

The average daily traffic volume was determined at each of the four monitoring sites. Based on the midpoint of each road, six points at a distance of 100, 200, and 300 m were selected on each side of each road. The number of cars passing each point was counted over 14 consecutive days (24 h a day), and the results were then averaged.

### 2.3. Chemical Analysis

Following exposure, the moss samples were removed from the nylon net and dried to a constant weight in a thermostat oven at 80 °C for 12 h, ground into powder, and kept in a clean, dry plastic bag. For chemical analysis, 0.5 g of the moss sample was digested in a solution of HCLO_4_:HNO_3_ = 1:4 for approximately 12 h. The filtered solution was then dried to a white powder on an electric iron plate. The powder was dissolved in distilled water up to 25 mL in a volumetric flask for chemical analysis. Two parallel samples were prepared for each moss sample. All concentrations were reported as mean values on a dry weight basis (mg·kg^−1^).

The concentrations of five heavy metals (Cr, Cu, Pb, V, and Zn) in each moss sample were determined by the Inductively Coupled Plasma-Atomic Emission Spectrometry (ICP-AES) (Perkin Elmer Corporation, Rodgau, Germany) method [37].

### 2.4. Statistical Analysis

To assess the accumulation of each heavy metal by *S. junghuhnianum*, relative accumulation factors (RAF) were calculated using Equation (1):

(1) $$\mathrm{RAF}=\left(C_{\mathrm{exposed}}-C_{\mathrm{initial}}\right)/C_{\mathrm{initial}}$$

where $C_{\mathrm{exposed}}$ was the content of each element after exposure and $C_{\mathrm{initial}}$ was the content before exposure.

Contaminations factors (CF), used to assess the degree of anthropogenic influence, were calculated using Equation (2):

(2) $$\mathrm{CF}=C_{\mathrm{moss}}/C_{\mathrm{background}}$$

where $C_{\mathrm{moss}}$ is the content of the element in the moss samples after exposure, and $C_{\mathrm{background}}$ is the content of the same element determined before exposure. The scale for CF categories proposed by Ma et al. [38] was applied in the present study. This comprises five categories: (1) C1: CF ≤ 1.2, no contamination; (2) C2: 1.2 < CF ≤ 2.2, slight contamination; (3) C3: 2.2 < CF ≤ 3.3, moderate contamination; (4) C4: 3.3 < CF ≤ 4.3, severe contamination; (5) C5: CF > 4.3, extreme contamination.

The correlations between the concentrations of Cr, Cu, Pb, V, and Zn in the moss and traffic volume were analyzed by the correlative analysis method using SPSS 17.0 (IBM Corporation, Somers, NY, USA). The statistical significance was based on 0.05.

## 3. Results and Discussion

### 3.1. Concentrations of Cr, Cu, Pb, V, and Zn in Moss before and after Exposure

**Figure 2:**
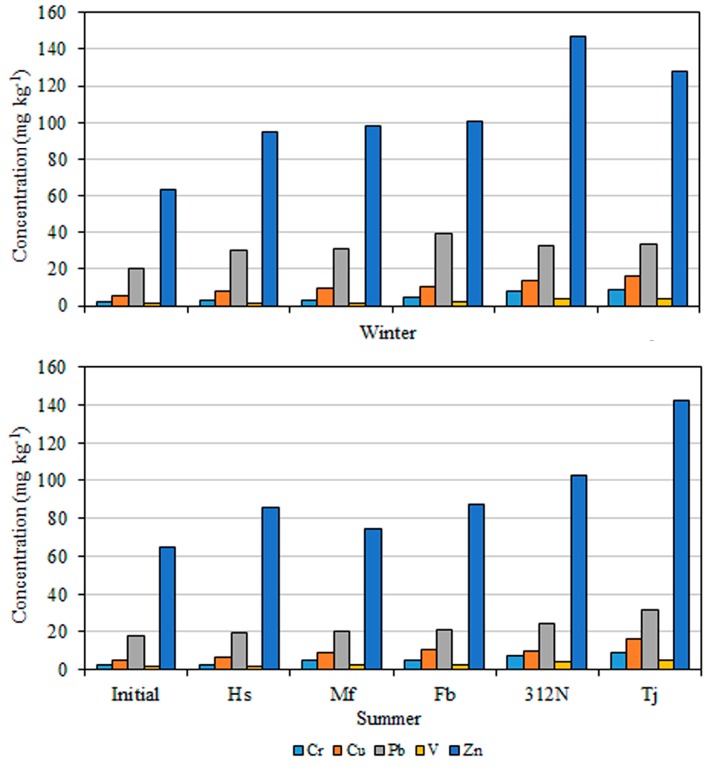
Average concentration of elements (mg·kg^−1^) in *Sphagnum junghuhnianum* exposed to air in bags for 6 weeks at Huishan (Hs), Minfeng Road (Mf), Fengbin Road (Fb), 312 National Road (312N), and Tongjiang Road (Tj).

The average concentrations of Cr, Cu, Pb, V, and Zn in moss bags after 6 weeks of exposure (Table 1) were used to compare the levels of atmospheric pollution at the different sample sites. The concentrations of all elements in the exposed moss bags were higher than the initial concentrations. As shown in Table 1, in the winter, following 6 weeks of exposure, the concentrations of Cr were 2.55–8.4 mg·kg^−1^ similarly, the concentrations of Cu, Pb, V, and Zn were 7.83–16, 30.05–39.58, 1.68–4.03, and 94.78–146.7 mg·kg^−1^, respectively. Moss sample analyses revealed a concentration of Cr in the range of 3.0–8.75 mg·kg^−1^ following exposure during the summer, with markedly different concentrations of Cu, Pb, V, and Zn (6.84–16.3, 19.85–31.65, 1.98–5.03, and 74.55–142.1 mg·kg^−1^, respectively). In general, an increasing level of pollution is observed from Huishan to Minfeng, Fengbin, 312 National Road, and Tongjiang Avenue (Figure 2). Therefore, according to the mean metal concentrations reported in Table 1, Tongjiang Avenue was the most polluted site, with the highest values for all elements, except for Zn recorded in winter in 312 National Road (146.7 versus 127.63). Moreover, it is worth noting that the control site Huishan had a pollution level that was comparable to Minfeng Road for Pb, V, and Zn. This unexpected result could depend on the long-range transport of heavy metals [39].

**Figure 3:**
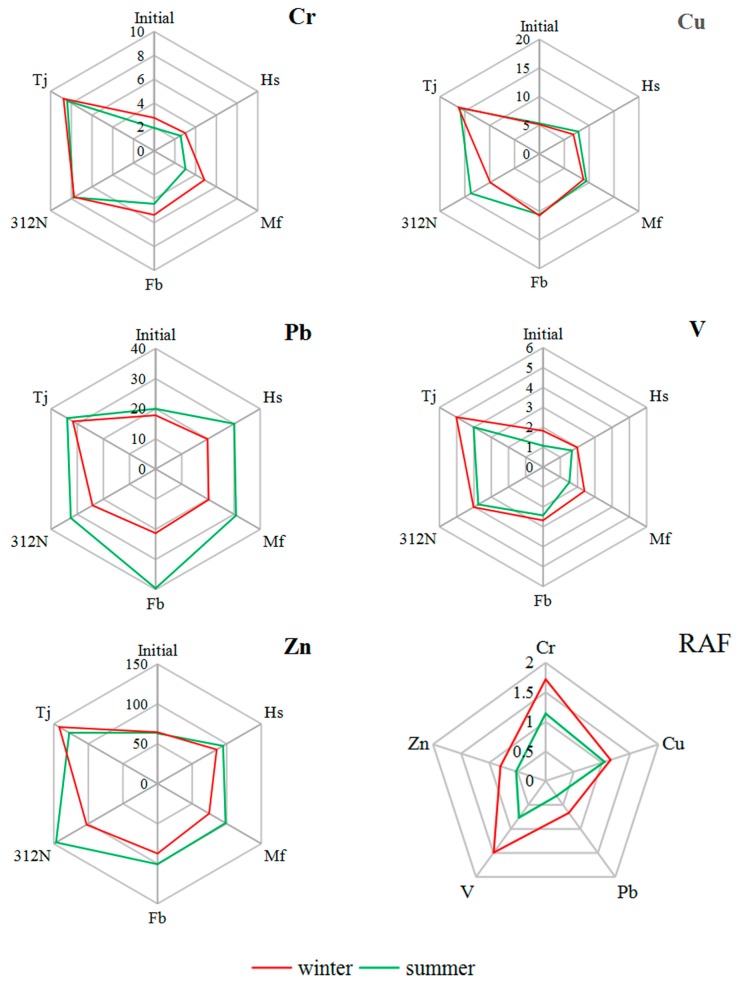
Seasonal variation in metal concentrations (mg·kg^−1^) in the moss *S. junghuhnianum* exposed to air in bags for 6 weeks at five monitoring sites as indicated by the average relative accumulation factor (RAF) for each metal in winter and summer.

The mean concentrations after 6 weeks of exposure and net increases over the winter and summer at the five different sampling sites are shown in Figure 2 and Figure 3. Compared with the initial content, the concentration of Zn varied widely. In addition, the highest concentrations of Cr, Cu, Pb, V, and Zn were recorded on Tongjiang Avenue during the summer, the high concentrations of Cr, Cu, and V occurred on Tongjiang Avenue during the winter, and the highest concentrations of Pb and Zn during the winter were recorded in Minfeng and 312 National Road, respectively. The lowest values of Cr, Cu, Pb, V, and Zn were recorded in Minfeng during both winter and summer. Tongjiang Avenue is an important traffic thoroughfare with a large vehicle flow, followed by 312 National Road and Fengbin. Therefore, the pollution of Tongjiang Avenue is the most serious. By contrast, Minfeng connects several residential areas with a relatively small traffic volume and relatively slight contamination. The concentrations of elements in the moss bags were higher than those in the control site, indicating that they were all polluted by different levels of heavy metals. The degree of pollution was as follows (in descending order): Tongjiang Avenue, 312 National Road, Fengbin, Minfeng, and Huishan (Figure 2). These findings support the hypothesis that vehicles are the main source of these metals. The highest concentration of metals in the moss bags was of Zn, followed by Pb, Cu, Cr, and V (Figure 2).

### 3.2. Relative Accumulation Factors and Contamination Factors of Metals in the Moss Samples

#### 3.2.1. Relative Accumulation Factors

Table 1 presents the RAFs of the metals in the *S. junghuhnianum* moss samples at the five monitoring sites after 6 weeks exposure. An RAF avoids the effect of the initial element concentration on the final concentration, and has been previously used to compare the accumulation of metals by different monitoring species [9]. During the winter monitoring period, the average RAFs of the heavy metals were Cr: 1.72, Cu: 1.15, Pb: 0.67, V: 1.49, and Zn: 0.80. Thus, the most-accumulated element in the moss bags was Cr, followed by V > Cu > Zn > Pb. During the summer monitoring period, the average RAFs were Cr: 1.14, Cu: 1.05, Pb: 0.31, V: 0.76, and Zn: 0.52, resulting in the order Cr > Cu > V > Zn > Pb. It can be seen from Figure 3 that the average RAFs of the five heavy metal elements in the winter were higher than those in the summer, indicating that air pollution is more serious in the winter than in the summer. Previous studies have reported that the amount of metal that accumulates in moss bags is related to the associated weather conditions [40]. At the study site, there is less precipitation during the winter compared with the summer and, therefore, pollutants in the atmosphere are less likely to be washed out of the atmosphere, to settle on and be absorbed by vegetation, with the reverse being true in the summer. Consequently, the summer pollution is lighter than in the winter due to more precipitation in summer than in winter.

In previous studies, an RAF higher than 0.5 indicated a slight elemental enrichment in moss, and values >1 indicated considerable elemental enrichment [33,41]. In the current study, the RAF indicated Cr, Cu, and V to be the most abundant elements in the moss exposed to air within the studied roads.

#### 3.2.2. Analysis of Contamination Factors and Pollution Levels

The results of the CF analysis are given in Table 1. During the winter, the mean CFs were Cr: 2.32, Cu: 1.59, Pb: 1.14, V: 1.75, and Zn: 1.25, indicating the order of the heavy metals in terms of average CF across the monitoring sites to be: Cr > Cu > V > Zn > Pb. During the summer, the CFs were Cr: 2.22, Cu: 1.68, Pb: 1.23, V: 1.79, and Zn: 1.19, indicating the order of the heavy metals in terms of average CF across the monitoring sites to be: Cr > V > Cu > Pb > Zn. Thus, in both monitoring periods, Cr has the highest CF, indicating that atmospheric pollution with Cr to be the most serious of all metals investigated and, thus, requiring the most urgent attention.

Based on the CF results, pollution levels are shown in Table 2. The monitoring sites were characterized by three categories of contamination scales: C1, C2, and C3, described as no pollution, slight pollution, and moderate pollution, respectively. During the winter, the levels of Cr, Pb, V, and Zn in Minfeng indicated no pollution, whereas those of Cu indicated only slight pollution; by contrast, the levels of Cr, Cu, Pb, and V in Fengbin indicated slight pollution, whereas those of Zn indicated no pollution. The level of Cr and V in 312 National Road indicated moderate pollution, those of Cu and Zn indicated slight pollution, and that of Pb indicated no pollution; the level of pollution at Tongjiang Avenue was similar to that of 312 National Road. During the summer, the levels of Cr, Cu, and V in Minfeng indicated slight pollution, whereas those of Pb and Zn indicated no pollution; the Fengbin pollution level was consistent with that of Minfeng. By contrast, the level of Cu, Pb, and V in 312 National Road indicated slight pollution, that of Cr indicated moderate pollution, and that of Zn indicated no pollution. Finally, the level of Cr, Cu, and V in Tongjiang Avenue indicated moderate pollution, whereas those of Pb and Zn indicated slight pollution. In summary, the CF values for heavy metals were higher compared with other roads, except for Zn in winter in 312 National Road (1.55 versus 1.35), and the differences in CF values suggest a high risk of heavy metal contamination in Tongjiang Avenue.

### 3.3. Correlation Analysis between Metal Concentrations and Traffic Volume

**Figure 4:**
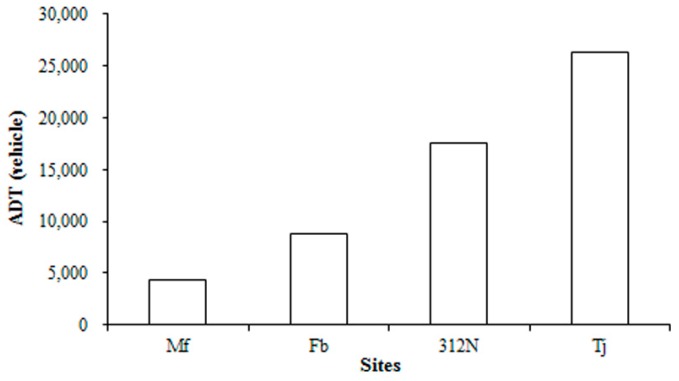
Traffic statistics for each of the study sites (Mf: Minfeng Road, Fb: Fengbin Road, 312N: 312 National Road, Tj: Tongjiang Avenue) ADT: average daily traffic volume.

Traffic volume statistics are shown in Figure 4. The traffic volume order was: Tongjiang Avenue (26,310) ˃ 312 National Road (17,490) ˃ Fengbin (8730) ˃ Minfeng (4345), whereas the order of the study sites in terms of average heavy metal concentration was: Tongjiang Avenue ˃ 312 National Road ˃ Fengbin ˃ Minfeng ˃ Huishan, indicating that the heavy metal content in the moss bag was positively correlated with the traffic volume; that is, the higher the traffic volume, the higher the heavy metal content and the more serious the level of pollution. Huishan is a scenic spot, far away from traffic pollution; thus, it was unsurprising that it had the lowest concentration of heavy metals. Guttormsen [42] reported that an average daily traffic volume of 12,000 vehicles on both sides of a road resulted in serious levels of pollution within 20 m of that road. In this study, the average daily traffic volume of 312 National Road and Tongjiang Avenue was 17,490 and 26,310 vehicles, respectively. Thus, the levels of heavy metal pollution at these sites were the most serious. The pollution source from vehicle movements can be summarized as [23]: (1) exhaust emissions from the tailpipe; (2) non-exhaust emissions due to the wear and tear of vehicle parts, such as brakes, tyres, and clutch; and (3) resuspension of road dust. In previous studies, traffic emissions mainly contributed to increased concentrations of Cr, Cu, Pb, V, and Zn, whereas fuel combustion (known as “exhaust emissions”) was the major source of Cr, Cu, and V emissions [43]. Pb could originate from brake-wear-related emissions [44]. In addition, tire abrasion emissions and fuel combustion have been recognized as important sources of Zn [45].

The results of the correlation analysis between heavy metal concentration and average daily traffic volume are provided in Table 3. There was a significant positive correlation (*p* < 0.05) between the concentrations of Cr, Cu, and V and average daily traffic volume during the winter, but not between Zn and Pb and the average daily traffic volume. During the summer, there was a significant positive correlation (*p* < 0.05) between Cr, Pb, V, and Zn and average daily traffic volume. This result does not completely agree with the literature in fact, in a previous study [46], the authors found a significant correlation between tyre-wear Zn emission and daily traffic volume, but the Zn level was 1000-fold higher than that of Cu, whereas in this study Zn concentration was about 10-20-fold higher than Cu. There was a negative correlation between Pb and average daily traffic volume in winter; this could be related to the long residence time of Pb in the atmosphere. There was in general a significant correlation between the concentration of heavy metals and the average daily traffic volume, indicating that traffic volume has a direct impact on heavy metal concentrations.

### 3.4. Correlation Analysis between Metal Elements

**Figure 5:**
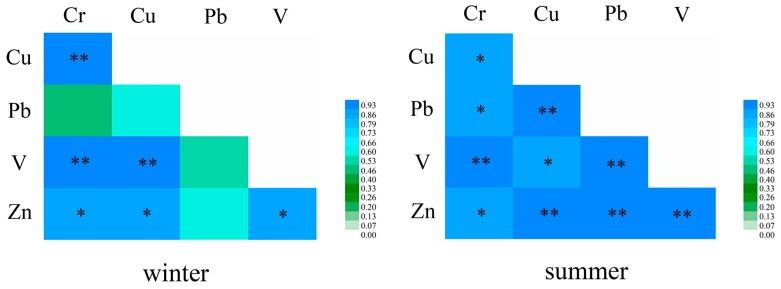
Pearson correlation coefficient matrix between heavy metals in moss bags in winter and summer. * Correlation significant at the 0.05 level; ** Correlation significant at the 0.01 level.

The Pearson’s correlation coefficients of heavy metals in the five monitoring sites are summarized in Figure 5. During the winter, the correlation coefficients were higher than 0.960 for Cu–Cr, V–Cr, and V–Cu, and were between 0.906 and 0.913 for Zn–Cr, Zn–Cu, and Zn–V. During the summer, the correlation coefficients were higher than 0.920 for Pb–Cu, V–Cr, V–Pb, Zn–Cu, Zn–Pb, and Zn–V, and between 0.871 and 0.906 for Cu–Cr, Pb–Cr, V–Cr, and Zn–Cr. The very high coefficients between these pairs of elements suggest that they had a common source, namely from automobile exhausts, vehicle parts, tyre wear and tear, and friction release when braking. There was no significant correlation between Pb and all of the other elements in winter. This could be related to the deposition of Pb as a result of its physicochemical characteristics, which result in a long residence time in the atmosphere.

## 4. Conclusions

In this study, heavy metals accumulated in *S. junghuhnianum* moss bags were exposed to air for 6 weeks at different sites across Wuxi city. Higher concentrations of metals were recorded in moss bags placed in Tongjiang Avenue. The RAF generally showed that pollution was heavier in winter than in summer. Significant correlations were found between metal concentrations and traffic volume in winter (Cr, Cu, V, Zn) and summer (Cr, Pb, V, Zn). The correlation between the different heavy metals showed that they had a common source: traffic pollution. The CF for Cr in the moss bags was the highest, indicating that Cr is the most serious pollutant. The concentrations of metals in *S. junghuhnianum* significantly reflected the degree of atmospheric pollution. Thus, the moss bag monitoring technique can be used to monitor heavy metal pollution in urban areas. However, using uniformly cloned mosses could result in more accurate survey data that can be compared across studies [19,47].

## Figures and Tables

**Table 1:** The concentration of metals (mg·kg^−1^, dry weight) in the moss bags over 6 weeks during the winter and summer.

Parameter	Cr		Cu		Pb		V		Zn	
	Winter	Summer	Winter	Summer	Winter	Summer	Winter	Summer	Winter	Summer
Initial	1.93	2.78	5.35	5.15	20.0	17.85	1.08	1.83	63.05	64.55
SD	0.18	0.81	0.07	0.21	0.57	1.34	0.04	0.18	0.99	1.63
Huishan										
Mean	2.55	3.0	7.83	6.84	30.05	19.85	1.68	1.98	94.78	85.55
SD	0.21	0.07	0.67	0.35	2.83	0.07	0.11	0.04	7.60	1.06
RAF	0.32	0.08	0.46	0.33	0.50	0.11	0.56	0.08	0.50	0.33
Minfeng Road										
Mean	3.03	4.85	9.45	8.88	30.75	20.3	1.53	2.4	98.3	74.55
SD	0.74	0.07	1.84	0.81	0.21	0.07	0.32	0.14	0.28	3.75
RAF	0.57	0.75	0.77	0.72	0.54	0.14	0.42	0.32	0.56	0.16
CF	1.19	1.62	1.21	1.30	1.02	1.02	0.91	1.22	1.04	0.87
Fengbin Road										
Mean	4.43	5.35	10.58	10.75	39.58	21.28	2.43	2.68	100.63	87.28
SD	0.11	0.21	0.32	1.20	1.87	2.79	0.18	0.39	1.24	2.79
RAF	1.30	0.93	0.98	1.09	0.98	0.19	1.26	0.47	0.60	0.35
CF	1.74	1.78	1.35	1.57	1.32	1.07	1.45	1.35	1.06	1.02
312 National Road										
Mean	7.78	7.73	13.75	9.9	32.45	24.13	3.75	4.03	146.7	102.48
SD	1.03	0.88	0.85	1.56	3.32	0.04	0.35	0.88	11.6	24.01
RAF	3.04	1.78	1.57	0.92	0.62	0.35	2.49	1.21	1.33	0.59
CF	3.05	2.58	1.76	1.45	1.08	1.22	2.24	2.04	1.55	1.20
Tongjiang Avenue										
Mean	8.4	8.75	16	16.3	33.8	31.65	4.03	5.03	127.63	142.1
SD	0.14	0.07	0.21	1.20	0.92	0.07	0.11	0.11	3.22	14.71
RAF	3.36	2.15	1.99	2.17	0.69	0.77	2.74	1.75	1.02	1.20
CF	3.29	2.92	2.05	2.39	1.13	1.59	2.40	2.54	1.35	1.66
RAF_mean_	1.72	1.14	1.15	1.05	0.67	0.31	1.49	0.76	0.80	0.52
CF_mean_	2.32	2.22	1.59	1.68	1.14	1.23	1.75	1.79	1.25	1.19

Mean: average value; SD: standard deviation; RAF_mean_ = relative accumulation factor (RAF); CF_mean_ = contamination factor (CF).

**Table 2:** The contamination factors (CFs) and contamination classification.

Parameter	Cr		Cu		Pb		V		Zn	
	Winter	Summer	Winter	Summer	Winter	Summer	Winter	Summer	Winter	Summer
Minfeng Road										
CF	1.19	1.62	1.21	1.30	1.02	1.02	0.91	1.22	1.04	0.87
Classification	C1	C2	C2	C2	C1	C1	C1	C2	C1	C1
Contamination	N	S	S	S	N	N	N	S	N	N
Fengbin Road										
CF	1.74	1.78	1.35	1.57	1.32	1.07	1.45	1.35	1.06	1.02
Classification	C2	C2	C2	C2	C2	C1	C2	C2	C1	C1
Contamination	S	S	S	S	S	N	S	S	N	N
312 National Road										
CF	3.05	2.58	1.76	1.45	1.08	1.22	2.24	2.04	1.55	1.20
Classification	C3	C3	C2	C2	C1	C2	C3	C2	C2	C1
Contamination	M	M	S	S	N	S	M	S	S	N
Tongjiang Avenue										
CF	3.29	2.92	2.05	2.39	1.13	1.59	2.40	2.54	1.35	1.66
Classification	C3	C3	C2	C3	C1	C2	C3	C3	C2	C2
Contamination	M	M	S	M	N	S	M	M	S	S

N: no contamination; S: slight contamination; M: moderate contamination.

**Table 3:** Correlation analysis between the concentration of heavy metals and traffic volume.

Pearson Correlation					
	Cr	Cu	Pb	V	Zn
ADT in winter	0.964 *	0.998 *	−0.068	0.956 *	0.750
ADT in summer	0.981 *	0.863	0.964 *	0.993 *	0.982 *

ADT: Average daily traffic volume * Correlation is significant at the 0.05 level.
